# Genetic Determinants of T-Cell Homeostasis in Critical Illness: An Exploratory Analysis of Immune Gene Variants and TREC Dynamics

**DOI:** 10.3390/jpm16060278

**Published:** 2026-05-23

**Authors:** Alesya S. Gracheva, Darya A. Kashatnikova, Maryam B. Khadzhieva, Vladislav E. Zakharchenko, Tatyana N. Krylova, Artem N. Kuzovlev, Lyubov E. Salnikova

**Affiliations:** 1 Vavilov Institute of General Genetics, Russian Academy of Sciences, 119333 Moscow, Russia; palesa@yandex.ru (A.S.G.); dkashatnikova@vigg.ru (D.A.K.); 2 Federal Research and Clinical Center of Intensive Care Medicine and Rehabilitology, 107031 Moscow, Russia; mkhadzhieva@fnkcrr.ru (M.B.K.); vzakharchenko@fnkcrr.ru (V.E.Z.); tkrylova@fnkcrr.ru (T.N.K.); artem_kuzovlev@fnkcrr.ru (A.N.K.); 3 Lopukhin Federal Research and Clinical Center of Physical-Chemical Medicine, Federal Medical Biological Agency, 119435 Moscow, Russia; 4 National Research Center of Pediatric Hematology, Oncology and Immunology, 117997 Moscow, Russia

**Keywords:** traumatic brain injury (TBI), chronic critical illness (CCI), T-cell receptor excision circles (TREC) trajectory, whole-exome sequencing (WES), immune genes, qualifying genetic variants

## Abstract

**Background:** Chronic critical illness (CCI) following acute brain injury involves persistent immune dysfunction, yet its genetic determinants remain unclear. We investigated whether the rate of T-cell receptor excision circle (TREC) depletion—a proposed marker of adaptive homeostatic resilience—is associated with the burden of rare damaging genetic variants. **Methods:** Whole-exome sequencing (WES) was performed on a cohort of 84 patients (64 with traumatic brain injury, 20 with stroke). In a longitudinal sub-cohort (*n* = 27), patients were stratified into quartiles (Q1–Q4) based on the slope of their TREC trajectories. “Qualifying variants” (QVs) were defined using strict rarity (gnomAD allele frequency ≤ 0.001) and pathogenicity criteria. Gene-level burden (collapsing) analysis and permutation-based statistical testing (10,000 iterations) were employed to evaluate genetic enrichment in the extreme quartiles. **Results:** While baseline TREC levels were strictly age dependent (*p* < 0.0001), the rate of change (TREC slope) was age independent. Rapid TREC decline (Q1) correlated with significantly higher final SOFA scores (*p* = 0.001) and neutrophil-to-lymphocyte ratios (*p* = 0.020). Rare variant burden analysis revealed that Q1 patients were significantly more likely to harbor QVs in immune-related genes compared to the Q4 recovery group (odds ratio = 8.25; permutation *p* = 0.016). Patients with rapid decline were enriched for QVs in putative core “housekeeping” pathways essential for T-cell maintenance and DNA repair (e.g., *ERCC3*, *FANCM*), whereas variants in recovering patients were restricted to peripheral effector or structural pathways. **Conclusions:** Our findings suggest, as a conceptual framework, that an individual’s ability to maintain T-cell homeostasis during critical illness is influenced by their underlying genetic buffering capacity. We propose a hypothetical “two-hit” framework where physiological stress unmasks pre-existing fragilities in core homeostatic pathways—potentially reflecting a state of functional haploinsufficiency under extreme proliferative demand—leading to accelerated immune exhaustion. These results position the TREC slope as a dynamic, age-independent biomarker of genomic resilience in the ICU. All findings are exploratory and hypothesis generating.

## 1. Introduction

The host immune response is a critical determinant of outcomes in critical illness, including conditions such as sepsis and acute respiratory distress syndrome (ARDS). Severe physiological stress can trigger profound immune dysregulation characterized by simultaneous hyper-inflammation and immunosuppression, contributing significantly to morbidity and mortality [1,2]. While clinical and demographic factors provide some prognostic value, a substantial portion of the variability in patient trajectories remains unexplained, suggesting a pivotal role for underlying genetic predispositions [3,4].

T-cell receptor excision circles (TRECs) are DNA byproducts of T-cell receptor rearrangement in the thymus, and their quantification in peripheral blood serves as a biomarker for recent thymic output and the homeostatic stability of the naive T-cell compartment [5,6]. TREC levels decrease with age and are linked to chronic conditions like immunodeficiencies, autoimmune diseases, cardiovascular diseases, and renal failure [7,8,9]. TREC levels also have potential as a prognostic tool for nosocomial and community-acquired infections [10,11,12]. In critically ill patients, a decline in TREC levels has been associated with deep-seated immunosuppression and adverse outcomes [11,13,14].

Recently, we demonstrated that, in a cohort of 48 patients with moderate-to-severe traumatic brain injury (msTBI), lower absolute TREC levels correlated with older age and increased clinical severity; however, individual trajectories varied significantly [15]. Specifically, patients of the same age can exhibit diametrically opposed trends—some showing robust restoration and others rapid decline—indicating that the dynamic response to injury is not a simple function of chronological age. This clinical observation suggests that host-specific resilience, potentially rooted in genetic factors, dictates the stability of the T-cell compartment during critical illness.

Identifying genetic variants that impair such immune resilience could provide novel insights into disease pathogenesis and improve patient stratification. While common variants have been explored in genome-wide association studies [16,17], rare, highly penetrant “qualifying variants” (QVs) that disrupt the function of key immune genes represent a compelling but understudied source of risk [18].

In this exploratory hypothesis-generating study, we investigated the impact of rare predicted-damaging QVs on the longitudinal dynamics of TRECs in patients with acquired brain injury. We hypothesized that an accumulation of damaging QVs in immune-related genes might be associated with an accelerated decline in TREC levels, which could reflect an impaired capacity for homeostatic resilience.

## 2. Materials and Methods

### 2.1. Study Cohort and Clinical Data Collection

This observational study enrolled 84 unrelated patients of European ancestry (67 men, 17 women; median age 45 years, IQR: 33.5–60.8). From June 2023 to March 2024, participants were recruited from the Federal Research and Clinical Center of Intensive Care Medicine and Rehabilitology (FRCC ICMR). Participants included cases of moderate-to-severe subacute or chronic traumatic brain injury (msTBI; *n* = 64) or stroke (*n* = 20). Stroke and TBI cases were combined for genetic analysis, as both represent acute brain injury triggers that share common pathways of systemic immune-inflammatory response and stress-induced lymphodepletion. The cohort comprised 50 patients from a previous TBI study [19] and 34 newly recruited patients. Exclusion criteria included age < 18 years; developmental or hereditary genetic disorders; pregnancy; terminal-stage chronic disease; and military or substance-related injuries.

Whole-exome sequencing (WES) data were available for all 84 patients. TREC levels were measured in a subgroup of 41 TBI patients (age ≤ 60 years). Longitudinal data (≥2 measurements) were collected for 27 of these patients (TREC sub-cohort). Clinical status was tracked from admission to discharge or death, with additional sampling triggered by clinical deterioration (sepsis, organ dysfunction, neurological decline) or significant recovery (weaning from ventilation, restored consciousness). The study was approved by the FRCC ICMR’s local ethics committee (No. 2/23/4), and informed consent was obtained from all participants or their representatives.

### 2.2. Clinical Assessment

Severity was assessed using the Sequential Organ Failure Assessment (SOFA), Glasgow Coma Scale (GCS), and Disability Rating Scale (DRS). Organ failure was defined as a SOFA score ≥ 3 for any system. GCS was used for acute consciousness assessment (severe TBI: 3–8; moderate TBI: 9–12), while the DRS (range 0–29) was employed to track functional impairment and long-term recovery [20].

### 2.3. TREC Quantification

TREC levels in whole venous blood were measured using a previously described multiplex real-time PCR assay [12]. Genomic DNA was extracted from 200 µL of blood. The assay targeted the δREC-ψJα T-cell receptor (TREC) fragment, using the human albumin gene as an internal reference for normalization. Amplification was performed on a CFX96 instrument (Bio-Rad, Hercules, CA, USA). Absolute quantification was achieved using standard calibration curves derived from plasmid constructs. TREC levels were expressed as the number of copies per 100,000 nucleated cells, calculated with the formula: copies = [mean TREC copies/(mean albumin copies/2)] × 100,000.

### 2.4. Whole-Exome Sequencing and Bioinformatic Processing

The WES and variant calling methodologies are thoroughly delineated in [19]. Genomic DNA was isolated from blood using the QIAamp DNA Blood Mini Kit (Qiagen GmbH, Hilden, Germany). Sample sequencing was conducted at “Eugene” LLC (Moscow, Russia). DNA was fragmented, and libraries were prepared using the Agilent SureSelect Human All Exon v8 kit (Agilent Technologies, Santa Clara, CA, USA) for exonic enrichment. Sequencing was performed on the MGISEQ-2000 platform (MGI Tech Co., Ltd., Shenzhen, China). The bioinformatic pipeline included alignment to the GRCh38 reference genome (bwa mem2 v2.2.1); quality control (Picard v2.22.4); variant calling (bcftools mpileup v1.9, Strelka2 v2.9.2); and annotation (ANNOVAR web version, https://annovar.openbioinformatics.org/en/latest/, accessed 17 June 2024). Final quality assessment was performed with MultiQC v1.14. A minimum coverage of 10× was required for variant calls, with an average sequencing depth of 91.81 ± 54.05.

### 2.5. Variant Filtration and Functional Annotation

Variants were annotated using ANNOVAR and Ensembl Variant Effect Predictor (v112). Allele frequencies were sourced from gnomAD v2.1.1, and clinical significance was determined using ClinVar (30 October 2025 release). “Qualifying variants” (QVs) were defined as rare variants—meeting both an internal cohort threshold (allele count ≤ 4 in the 84-patient cohort) and an external population threshold (gnomAD AF ≤ 0.001 or absent from the database)—and annotated as pathogenic or likely pathogenic (P/LP) in ClinVar. We further assessed pathogenicity using CADD v1.7 PHRED scores; a threshold of ≥20 represents the top 1% of the most deleterious variations [21]. Gene set enrichment analysis was performed via STRING (v12.5) and Enrichr (web version, https://maayanlab.cloud/Enrichr/, accessed 11 October 2025). To ensure objectivity and reproducibility in the definition of immune-related genes, we utilized the Human Phenotype Ontology (HPO) term “Abnormality of the immune system” (HP:0002715). This standardized approach avoids the bias of manual gene selection and focuses on a set of genes with established clinical relevance to immune dysfunction.

The stability of pathway-level inference was ensured by utilizing gene set collection thresholds (minimum size of 5 genes) validated for small-scale genetic discovery [22,23]. This approach leverages functional aggregation to increase statistical power and mitigate the limitations of small sample sizes, a strategy supported by established benchmarking of enrichment algorithms in low-N clinical cohorts [24,25].

### 2.6. Statistical Analysis

All statistical analyses were performed using R (v4.3.2) and Python (v3.11) with standard scientific libraries (SciPy, Statsmodels, Pingouin). Data normality was assessed using the Shapiro–Wilk test; as most parameters exhibited non-normal distributions, continuous data are presented as median and interquartile range (IQR). Differences between independent groups were evaluated using the Mann–Whitney U test. Associations between categorical variables were assessed using Fisher’s exact test.

#### 2.6.1. Longitudinal Trajectory Analysis

To quantify dynamic changes, individual patient trajectories (slopes) for TREC, neutrophil-to-lymphocyte ratio (NLR), C-reactive protein (CRP), and Sequential Organ Failure Assessment (SOFA) scores were calculated. Slopes were derived using ordinary least squares (OLS) linear regression on all available longitudinal measurements per patient, where the slope coefficient (beta) represents the rate of change over time. The use of a clinical timescale (days since hospitalization) was informed by previous linear mixed-effects modeling in this cohort confirming its superior fit for TREC dynamics [15].

#### 2.6.2. Correlation and Age Adjustment

Bivariate correlations between non-normally distributed variables were assessed using Spearman’s rank correlation (rho). To rigorously evaluate the independence of TREC dynamics from chronological age, partial Spearman’s rank correlations were employed, controlling for age as a continuous covariate. This approach ensures that the observed associations between TREC slopes and clinical outcomes (final SOFA, final NLR) are not confounded by the baseline age-related decline in thymic output.

#### 2.6.3. Multiple Testing and Visualization

The Benjamini–Hochberg method was used to control the false discovery rate (FDR) of multiple tests. A two-tailed *p* < 0.05 was considered statistically significant. Graphs were generated using the SRplot platform (https://www.bioinformatics.com.cn/srplot, accessed on 1 March 2026) and Matplotlib (Version 3.10.0).

#### 2.6.4. Rare Variant Burden and Sensitivity Analysis

To rigorously evaluate the association between rare genetic variation and TREC dynamics while accounting for the small sample size of the longitudinal sub-cohort, we employed a collapsing approach based on the Cohort Allelic Sum Test (CAST) [26,27]. This hypothesis-driven burden analysis focused on a single pre-specified gene set: “Abnormality of the immune system” (HP:0002715). All subsequent pathway analyses (GO, STRING, HPO term enrichment) were performed as exploratory hypothesis-generating analyses and are clearly labeled as such. Rare QVs (gnomAD AF ≤ 0.001, AC ≤ 4)) were aggregated within the “Abnormality of the immune system” gene set (HP:0002715) to create a single burden variable per quartile. The distribution of this genetic burden was compared between extreme quartiles (Q1 vs. Q4) using a two-tailed Fisher’s exact test. To further ensure the robustness of the findings and verify the stability of the clinical inference in our small cohort, we supplemented this with a permutation test (10,000 iterations) to calculate an empirical *p*-value by shuffling group labels [28]. Furthermore, Jackknife (leave-one-out) sensitivity analysis was conducted by systematically removing individual patients and recalculating the enrichment *p*-value to verify the stability of the inference and ensure results were not driven by outliers.

## 3. Results

### 3.1. Patient Characteristics

The demographic and clinical profiles of 84 patients (64 TBI, 20 stroke) are summarized in Supplementary Table S1. The cohort was stratified by baseline T-cell receptor excision circle (TREC) data availability: TREC-available (*n* = 41) and non-TREC (*n* = 43). All patients in the TREC-available group had a history of TBI and were significantly younger than the non-TREC group (median 40 vs. 59 years; *p* < 0.0001). While baseline neurological status (GCS, DRS), severity (SOFA), and inflammatory markers (NLR, CRP) were comparable after FDR correction, the non-TREC group exhibited a more severe clinical trajectory. This included significantly higher final SOFA scores and CRP levels (*p* < 0.005), plus a higher incidence of sepsis/septic shock (58.1% vs. 12.2%, *p* < 0.0001), likely reflecting their advanced age. Within the TREC-available group, clinical characteristics were similar between longitudinal (*n* = 27) and single-measurement (*n* = 14) patients, validating the cohort for longitudinal analysis. The overall population faced substantial systemic strain and fragility: (i) metabolic/hematologic—50.0% had anemia, and 39.29% had protein-energy malnutrition; (ii) interventions—76.19% required a tracheostomy, and only 21.43% required no medical devices; and (iii) complications—79.76% developed nosocomial pneumonia, and only 16.67% remained free of nosocomial infections throughout their stay. The longitudinal subgroup (*n* = 27) mirrored this high morbidity, with only 14.81% avoiding nosocomial infections.

### 3.2. Clinical Significance of TREC Dynamics and Correlation with Outcomes

**Figure 1:**
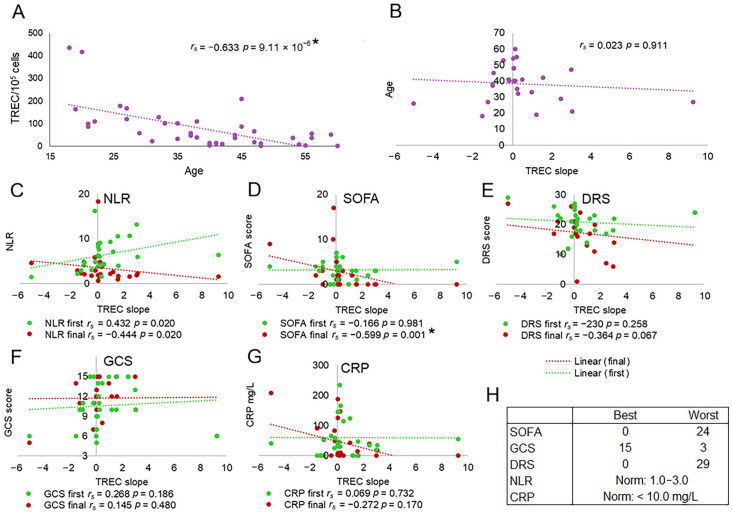
Age-dependency and clinical trajectories in relation to TREC dynamics. (**A**,**B**) Baseline TREC levels vs. age (*n* = 41) and TREC slope vs. age (*n* = 27). (**C**–**G**) Correlation of admission vs. final parameters with TREC slope for NLR, SOFA, DRS, GCS, and CRP. Panels (**C**,**D**) illustrate that rapid TREC depletion specifically associates with worsening systemic organ failure and inflammation. (**H**) Reference table for clinical scores and normal ranges. * Indicates FDR-corrected significance (*p* < 0.05). Abbreviations: CRP, C-reactive protein; DRS, Disability Rating Scale; GCS, Glasgow Coma Scale; NLR, neutrophil-to-lymphocyte ratio; SOFA, Sequential Organ Failure Assessment; TREC, T-cell receptor excision circle.

We first analyzed the relationship between T-cell homeostasis and age to establish clinical relevance. While baseline TREC levels showed the expected strong negative correlation with age, the TREC trajectory slope did not (*r_s_* = −0.02, *p* = 0.91); Figure 1A,B). This suggests that while the initial T-cell reservoir is age-dependent, the rate of depletion during critical illness is driven by age-independent factors. We then assessed if TREC dynamics serve as a surrogate for clinical stability (Figure 1C–H). TREC trajectory slopes showed evolving relationships with systemic inflammation. In the TREC sub-cohort, a higher baseline NLR positively correlated with the subsequent TREC slope (*r_s_* = 0.43, *p* = 0.020). This relationship later inverted. A steeper TREC decline (negative slope) significantly correlated with a higher final NLR (*r_s_* = −0.44, *p* = 0.020; Figure 1C) and higher final SOFA scores (*r_s_* = −0.6, *p* = 0.001; Figure 1D). Similar trends were observed for final DRS (Figure 1E), though GCS and CRP showed no associations (Figure 1F,G). This divergence highlights that while early inflammatory markers may foreshadow immune shifts, TREC depletion specifically mirrors escalating organ dysfunction and failed clinical recovery rather than the initial severity of the insult.

### 3.3. Patient Stratification by TREC Dynamics

**Figure 2:**
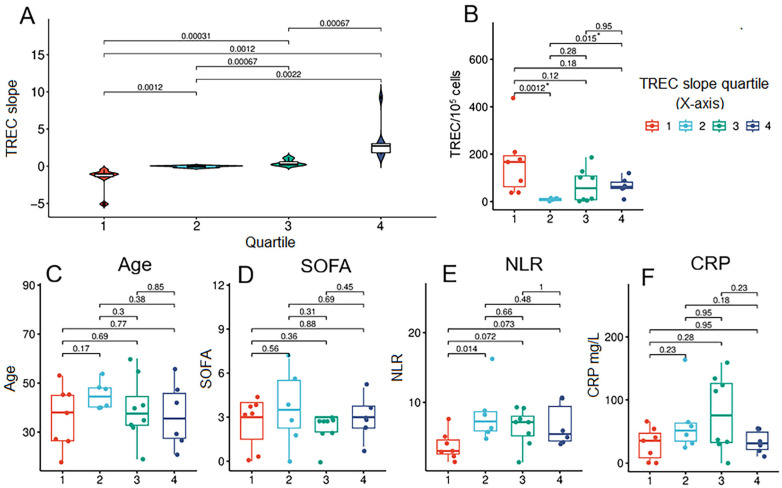
Patient stratification. (**A**) Violin plot of individual TREC slopes used for patient stratification into quartiles (Q1–Q4). All *p*-values are significant following the FDR correction. (**B**–**F**) Baseline characteristics by quartile: (**B**) TREC levels, (**C**) age, (**D**) SOFA score, (**E**) NLR, and (**F**) CRP. (**B**) * Indicates FDR-corrected significance (*p* < 0.05). Abbreviations: CRP, C-reactive protein; NLR, neutrophil-to-lymphocyte ratio; SOFA, Sequential Organ Failure Assessment; TREC, T-cell receptor excision circle.

To isolate the determinants of immune fragility, we stratified 27 patients with sequential data into quartiles (Q1–Q4; 7, 6, 8, and 6 patients) based on their TREC trajectory slopes (Figure 2A). By definition, slopes differed significantly across all quartiles in post hoc pairwise comparisons. Notably, age was similar across groups (Figure 2C), particularly between the extreme quartiles (Q1 median: 36; Q4 median: 35.5). Baseline clinical status—including SOFA (Figure 2D), NLR (Figure 2E), and CRP (Figure 2F)—also showed no significant differences among quartiles. However, baseline TREC levels differed significantly between the lowest (Q1) and second lowest (Q2) slope quartiles (Figure 2B).

### 3.4. Selection of Qualifying Variants from WES Data

**Figure 3:**
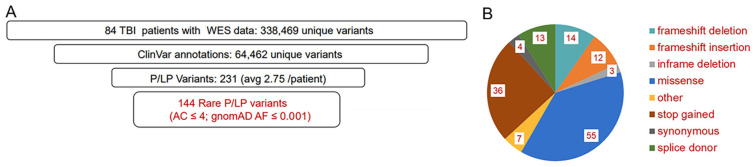
Selection and characterization of qualifying variants (QVs). (**A**) Variant selection workflow. (**B**) Functional consequences of QVs. Abbreviations: AC, allele count; AF, allele frequency; gnomAD, Genome Aggregation Database; P/LP, pathogenic/likely pathogenic; WES, whole-exome sequencing.

Having excluded age and primary clinical severity as main drivers of TREC dynamics, we next investigated whether genetic predispositions might account for these distinct trajectories. To define a set of functionally relevant “qualifying variants” (QVs), we first utilized the full cohort (*n* = 84) to compare the frequency of functional variants in our sample with literature data. Annotation of whole-exome sequencing (WES) data was performed using ClinVar (Figure 3A). We identified 231 pathogenic/likely pathogenic (P/LP) variants (averaging 2.75 per patient), consistent with established European population rates [29]. To establish a robust rarity threshold, we then applied filters based on both internal allele count (AC ≤ 4) and external population frequency (gnomAD AF ≤ 0.001). This filtering identified 144 rare P/LP candidates, designated as qualifying variants (QVs). These 144 QVs were primarily missense SNVs (38.0%), stop-gained SNVs (25.0%), frameshift deletions (9.7%), and splice donor variants (9.0%) (Figure 3B).

### 3.5. Exploratory Analysis Reveals That Damaging Variants in Immune Genes Are Associated with Accelerated TREC Decline

**Figure 4:**
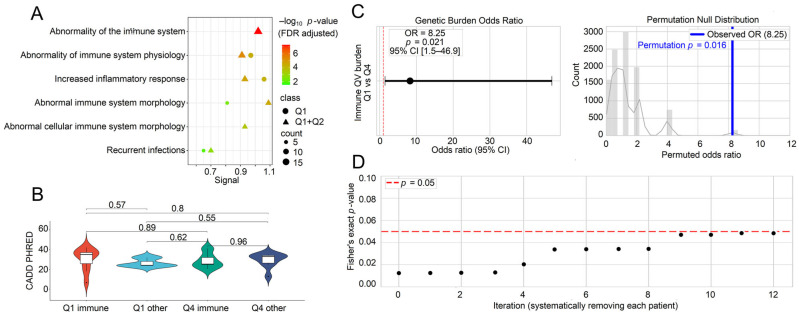
Genetic analysis of qualifying variants (QVs) in patients with extreme TREC dynamics. (**A**) Gene set enrichment analysis of genes harboring QVs in patients with TREC decline. (**B**) Predicted pathogenicity (CADD PHRED scores) of QVs across all TREC slope quartiles. (**C**) Rare variant burden analysis with forest plot showing odds ratio (OR) and 95% confidence interval for the burden of immune-related QVs (HPO:0002715) in the Q1 group compared to the Q4 group (**left panel**); null distribution of the odds ratio generated from 10,000 permutations; the blue line and the grey shaded area indicate the observed OR (8.25) with the 95% CI; and the permutation *p*-value (0.016) reflects the proportion of permuted values greater than or equal to the observed result (**right panel**). (**D**) Jackknife (leave-one-out) sensitivity analysis demonstrating stability of clinical inference. Each point represents a Jackknife iteration where one patient from the extreme groups (Q1 and Q4, *n* = 13 total) was systematically excluded, and Fisher’s exact *p*-value for immune QV enrichment was recalculated. The resulting distribution of *p*-values, all of which remain below the significance threshold (*p* < 0.05), confirms that the observed genetic association is a stable feature of the cohort and is not driven by the influence of any single outlier individual.

Gene set enrichment analysis (STRING) identified significant enrichment for the Monarch module within the Human Phenotype Ontology (HPO) (Supplementary Table S3). This and all subsequent GO/pathway analyses are exploratory and intended to generate hypotheses, not to provide confirmatory evidence. In patients with TREC decline (Q1 and Q1 + Q2), genes harboring QVs were significantly enriched for terms related to immune system abnormalities (Figure 4A). No such enrichment was found for the Q3 + Q4 or Q4 groups. By defining “immune genes” under the top HPO term “Abnormality of the immune system,” we compared CADD PHRED scores across quartiles but found no differences between Q1 and Q4 or between immune and non-immune genes (Figure 4B). Rare variant burden analysis, using the CAST-based collapsing approach, revealed that Q1 patients were significantly more likely to harbor damaging variants in genes related to immune system abnormalities compared to the Q4 group (OR = 8.25; 95% CI: 1.45–46.86; *p* = 0.021, Fisher’s exact test; *p* = 0.016, permutation test; Figure 4C). Given the small sample size, this odds ratio may be inflated, and the confidence interval is wide; the finding requires replication in an independent cohort. To address concerns regarding the small sample size, we performed a Jackknife sensitivity analysis, which demonstrated that the immune QV enrichment in Q1 remained stable and significant across iterations, confirming a collective genetic signal rather than an outlier-driven effect (Figure 4D).

After identifying the initial TREC dynamics, we conducted an exploratory analysis to determine if the observed genetic associations also applied to other clinical markers of inflammation (NLR and CRP) and organ dysfunction (SOFA). To ensure a rigorous assessment, we performed this analysis in two stages: first, a direct comparison within the TREC sub-cohort (*n* = 27) using matched timepoints (median of three measurements); and second, a validation analysis in the full cohort (*n* = 84) utilizing the complete longitudinal dataset (median measurements: NLR = 16, CRP = 15, SOFA = 7.5). Unlike the TREC analysis, the distribution of QVs among immune and other genes within each set was similar (Supplementary Figure S1). Notably, the absence of similar genetic enrichment for NLR, CRP, and SOFA reinforces the specificity of the findings. This marker-specific association suggests that the identified rare variants may not merely reflect a generalized burden of critical illness but could point to a selective vulnerability in pathways governing T-cell maintenance under stress.

### 3.6. Partial Correlations with Initial TREC Level, Controlling for Age

Partial correlation analysis adjusted for age (*n* = 41) revealed no significant relationships between initial TREC levels and admission severity, immune gene QV burden, or other inflammatory dynamics (all *p* > 0.05; Supplementary Table S4). These results suggest that dynamic changes (the slope) rather than the initial reservoir are the primary predictive factor.

### 3.7. Illustrative Cases: Rare P/LP Variants in Immune Genes in Patients with Extreme TREC Dynamics

**Figure 5:**
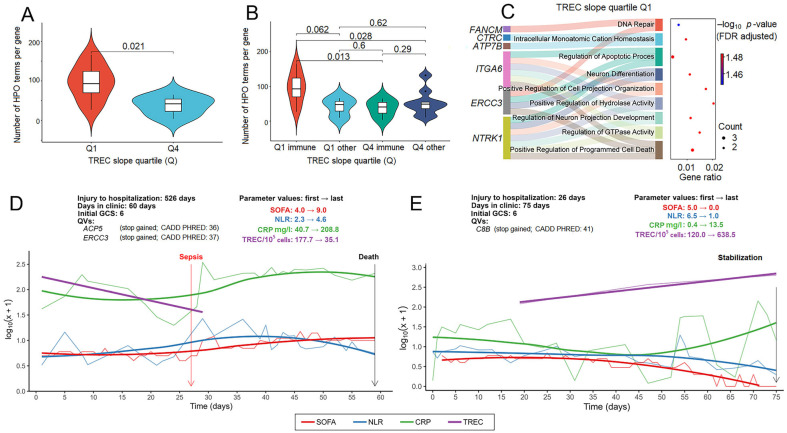
Genomic and clinical characterization of TREC Dynamics. (**A**) Violin plots comparing the density of HPO terms associated with genes in the Q1 vs. Q4 TREC trajectory groups. (**B**) Distribution of HPO term counts per gene, stratified by immune-related function and quartile (Q1 vs. Q4). (**C**) Sankey dot plot illustrating Gene Ontology (GO) biological processes enriched within the Q1 immune gene set. Connections represent the mapping of specific genes to functional categories. Enrichment was defined using a threshold of ≥2 gene per term and an FDR-adjusted *p* < 0.05 (Supplementary Table S5). Gene ratio indicates the proportion of queried genes relative to the total genes in the background set. (**D**,**E**) Clinical genetic summaries for representative patients from the Q1 (**D**) and Q4 (**E**) slope quartiles, highlighting the contrast between housekeeping and effector pathway variants. Abbreviations: CRP, C-reactive protein; GCS, Glasgow Coma Scale; HPO, Human Phenotype Ontology; NLR, neutrophil-to-lymphocyte ratio; QVs, qualifying variants; SOFA, Sequential Organ Failure Assessment; TREC, T-cell receptor excision circle.

To contextualize our statistical findings biologically, we examined gene characteristics in patients stratified by TREC trajectories. The following analyses are descriptive and hypothesis-generating; they are not intended for formal inference. The breadth of HPO terms associated with a gene serves as a proxy for its biological significance, specifically reflecting its pleiotropy and essentiality [30,31]. The phenotypic profiles of immune genes in the Q1 group were significantly enriched with HPO terms compared to those in Q4 (*p* = 0.021; Figure 5A). This enrichment was driven primarily by Q1 immune genes, which exhibited a higher number of HPO terms per gene than both Q4 immune genes and non-immune Q1 genes (*p* = 0.013 and trend of *p* = 0.062, respectively; Figure 5B). The diversity of phenotypic associations is corroborated by the wide range of significant biological processes governed by Q1 immune genes (Figure 5C; Supplementary Table S5). This visualization, restricted to terms containing ≥2 genes, serves to illustrate the functional themes concentrated in the rapid-decline group rather than to define definitive gene sets for subsequent inference.

We also performed a patient-based qualitative review of immune QVs (Supplementary Table S6). Biological functions and disease associations for the identified variants were annotated using the UniProtKB, GeneCards, and OMIM databases [32,33,34]. Most variants were heterozygous in genes typically associated with autosomal recessive conditions, suggesting that reduced gene dosage may become critical under the proliferative stress of critical illness—a state of proposed functional haploinsufficiency.

Beyond the quantitative enrichment of immune QVs in Q1 patients, a qualitative review revealed a functional dichotomy between the extreme quartiles (Supplementary Table S6). Q1 patients predominantly harbored defects in core “housekeeping” pathways essential for T-cell replication and systemic stability. These included genes critical for DNA repair (*FANCM*, *ERCC3*), homeostatic activation signaling (*ITGA6*, *NTRK1*), and cytokine regulation (*ACP5*). Conversely, immune variants in Q4 patients were restricted to effector pathways (e.g., complement component *C8B*) or tissue-specific structural genes (e.g., *NPHS2*, *CAPN3*). Clinically, Q1 patients experienced more severe conditions and higher peak SOFA scores. In contrast, Q4 patients demonstrated robust T-cell recovery despite nosocomial infections; notably, all Q4 patients achieved a final SOFA score of 0 and maintained a lower NLR than Q1 patients (Supplementary Figure S2). Two illustrative cases—Case 1 (Q1), with a multisystem DNA-repair defect, and Case 2 (Q4), with a localized effector variant—exemplify these contrasting trajectories (Figure 5D,E). This functional classification (housekeeping vs. effector) is proposed as a conceptual model to guide future studies, not as a proven biological distinction.

**Case 1:** Intrinsic Fragility and Proliferative Failure (Patient 105, Q1)

Patient 105, a 26-year-old male with severe TBI, exhibited a rapid TREC decline and carried two pathogenic stop-gain variants: *ACP5* and *ERCC3* (Figure 5D, Supplementary Table S6). *ACP5* encodes a negative regulator of interferon signaling, the deficiency of which likely primed a hyper-inflammatory state. Simultaneously, the *ERCC3* defect compromised DNA repair during the rapid T-cell division required for recovery. This “double hit”—systemic inflammation (CRP peak: 208.8 mg/L) paired with an intrinsic DNA-repair defect—created an environment where proliferative stress led to catastrophic T-cell collapse and a fatal outcome.

**Case 2:** Isolated Effector Defects and Immune Resilience (Patient 065, Q4)

In contrast, Patient 065, a 27-year-old female, exhibited recovering TREC dynamics (Q4) despite harboring a highly damaging stop-gain variant in *C8B* (Figure 5E). While *C8B* is essential for the membrane attack complex, it is a terminal effector protein mechanistically decoupled from the machinery of T-cell maintenance and lymphopoiesis. This case illustrates that variants in effector-only pathways do not inherently compromise T-cell resilience, allowing the adaptive immune system to remain robust even under the extreme physiological stress of critical illness.

Taken together, these illustrative cases demonstrate a pattern consistent with the idea that the clinical impact of rare immune variants may not be merely a function of their pathogenicity but could be fundamentally determined by whether the affected gene governs core “housekeeping” stability or peripheral “effector” tasks. This functional hierarchy offers a hypothetical explanation for why specific genomic architectures may predispose certain patients to immune collapse, while others maintain resilience, suggesting that TREC dynamics could serve as a clinical readout of an individual’s underlying genetic buffering capacity during critical illness.

## 4. Discussion

### 4.1. Principal Findings: Genetic Burden Is Linked to T-Cell Depletion

In this exploratory study, we demonstrate a specific association between the burden of rare damaging variants (QVs) in immune-related genes and the accelerated decline of T-cell receptor excision circles (TRECs) in critically ill patients. Our analysis revealed that individuals with the most rapid T-cell depletion (Q1) were significantly more likely to carry damaging variants in immune-centric genes (OR = 8.25; permutation *p* = 0.016). Given the small sample size, this association should be considered hypothesis-generating; the effect size is imprecise and likely inflated. Crucially, our clinical correlation analysis established that T-cell decline is not a bystander effect of aging or initial injury severity. While baseline TREC levels were strictly age dependent, the TREC trajectory slope was age independent. Furthermore, while admission scores (SOFA, NLR) did not predict the subsequent slope, the rate of TREC loss—specifically escalating organ failure and systemic inflammation—correlated with the dynamic failure of homeostatic resilience rather than a fixed baseline deficit.

### 4.2. A Proposed Hypothetical “Two-Hit” Framework of Genetic Fragility and Immune Collapse

We propose a hypothetical “two-hit” framework where acute physiological stress unmasks underlying genetic fragilities. The first hit involves the massive innate inflammatory surge following injury; while higher initial NLR generally associates with favorable recovery, the second hit occurs in individuals harboring rare variants in core “housekeeping” genes (e.g., *FANCM*, *ACP5*). In these susceptible patients, the extreme demand for T-cell proliferation may unmask a state that could be termed “stress-induced functional haploinsufficiency”, where a 50% reduction in gene dosage becomes rate-limiting [35]. This could lead to “abortive recovery” and accelerated T-cell depletion, evidenced by the late-phase inversion where steeper TREC decline correlates with worsening final NLR and SOFA scores. This model provides one possible molecular basis for the variable clinical expressivity often observed in inborn errors of immunity [36,37].

### 4.3. Critical Illness as a Catalyst for Accelerated T-Cell Aging

The depletion of TRECs in our cohort likely serves as a molecular signature of exhausted regenerative capacity. From a biological perspective, TREC levels reflect a dynamic equilibrium between thymic output and homeostatic peripheral proliferation. While the thymic contribution declines with age [38], peripheral maintenance relies on the division of existing cells, which effectively dilutes the TREC signal [39]. This process mirrors the naive-to-memory phenotype shift observed in immunological aging, where the loss of naive cells leads to a compartment dominated by differentiated cells with reduced clonal diversity and functional impairment [40,41,42].

Recent evidence indicates that critical illness can advance the intrinsic biological age of T-cells and leave lasting “transcriptional scars” that mimic advanced aging [43,44,45]. Our findings suggest that genetic variants in core pathways accelerate this “molecular clock.” Specifically, several identified genes participate directly in T-cell focused pathways: Integrin Alpha-6 (*ITGA6*) is essential for T-cell activation and vascular homing (GO:0042110), while *NTRK1* regulates proliferative signaling (UNIPROT: P04629). Variants in these genes, alongside DNA-repair bottlenecks (*ERCC3*, *FANCM*), likely impair the massive cellular expansion required for recovery, leading to “abortive proliferation” [46] and the accumulation of senescent-like phenotypes (e.g., Gzmk+, CD28− subsets) [47,48]. Thus, the TREC slope provides a window into an individual’s “genomic resilience”—the ability to maintain T-cell compartment stability in the face of acute perturbation, regardless of chronological age.

### 4.4. TREC Dynamics as a Clinical Readout of Homeostatic Resilience

The rapid fluctuations in TREC levels observed in patients with CCI [15] reflect a complex biological equilibrium between thymic output, peripheral proliferation, and stress-induced lymphocyte trafficking [49,50,51,52,53]. While transient declines are not inherently maladaptive [54,55], a persistent downward trajectory indicates a fundamental collapse of homeostatic resilience [56,57]. Our finding that the TREC slope is associated with specific genetic fragilities suggests that “genomic resilience” contributes to this equilibrium.

Clinically, TREC quantification offers a tool for real-time risk stratification: a “flexible” profile—where levels recover as inflammation subsides—suggests favorable resilience, whereas a “rigid” or persistently low profile may indicate T-cell sequestration, anergy, or apoptosis [58,59,60,61,62,63]. This aligns with the emerging paradigm of physical resilience versus physiological reserve [64,65], positioning the TREC slope as a proximal window into T-cell compartment stability. The superiority of longitudinal TREC slopes over static admission markers for predicting outcomes mirrors recent prognostic modeling in CCI [66], confirming that dynamic trajectories offer significantly higher predictive performance than traditional, “left-aligned” admission models.

### 4.5. Strengths and Limitations

The primary strength of this study is the shift from static baseline measurements to a dynamic analysis of T-cell homeostasis. Furthermore, our choice of a clinical timescale was informed by previous work in this cohort demonstrating that clinical time (days since hospitalization) is a more robust driver of TREC dynamics than biological time (days from injury), with no significant distortion introduced by the injury-to-hospitalization lag [16]. While the longitudinal sub-cohort was small (*n* = 27), this study was designed as an “Extreme Phenotype” analysis, comparing the most divergent clinical outcomes (Q1 vs. Q4). Such designs are highly effective for identifying rare variants with large effect sizes and allow for stable discovery in well-phenotyped cohorts [67,68]. To ensure statistical stability and mitigate the risk of false-positives, we employed gene-level burden analysis and permutation-based testing (10,000 iterations). A Jackknife sensitivity analysis further confirmed that the immune enrichment was a collective signal and not driven by individual outliers.

Despite these insights, several important limitations must be acknowledged. First, the sample size of the longitudinal sub-cohort (*n* = 27) is small, which limits statistical power and increases the risk of both false-positive and false-negative findings. Consequently, the observed odds ratio of 8.25 should be interpreted with extreme caution; estimates of effect size in rare variant studies with small samples are prone to inflation. Second, our findings are specific to the cohort studied: predominantly younger (median age 45 years), male, of European ancestry, and mostly with traumatic brain injury. Generalizability to older patients, other forms of critical illness (e.g., sepsis, ARDS, cardiac arrest), other ancestries, or pediatric populations is not established. Third, the proposed “two-hit” model and the classification of genes as “housekeeping” versus “effector” are conceptual and lack direct functional evidence in this clinical context. Forth, replication in independent, larger, multi-center cohorts is required before any clinical or mechanistic conclusions can be drawn.

### 4.6. Conclusions

In this exploratory hypothesis-generating study, a burden of rare damaging variants in immune-related genes was found to be associated with T-cell compartment stability in critically ill patients. This genetic predisposition may be specifically linked to the machinery of naive T-cell compartment stability rather than generalized inflammation. These findings provide a rationale for future genetic risk stratification in the ICU and suggest that restoring T-cell homeostasis could be a key therapeutic goal for susceptible individuals. While our results offer a biologically inspired “two-hit” framework for immune collapse, we emphasize that these mechanistic interpretations remain exploratory hypotheses. Given the small sample size, our proposed model serves as a conceptual foundation for future prospective validation in larger, multi-center cohorts.

## Data Availability

Data presented in this study are only available upon request from the corresponding author due to privacy or ethical restrictions.

## Supplementary Materials

The following supporting information can be downloaded at: https://www.mdpi.com/article/10.3390/jpm16060278/s1. Supplementary Figure S1. Genetic analysis of qualifying variants in patients with extreme NLR, CRP (mg/L), and SOFA dynamics (slope quartile 1 vs. quartile 4) in the TREC sub-cohort of 27 patients and in the full cohort of 84 patients. Abbreviations: CRP, C-Reactive Protein; NLR, Neutrophil-to-Lymphocyte Ratio; SOFA, Sequential Organ Failure Assessment. Supplementary Figure S2. Comparison of final discharge/death parameters in the five-panel analysis of Q1 vs. Q4 patients. Abbreviations: CRP, C-Reactive Protein; DRS, Disability Rating Scale; GCS, Glasgow Coma Scale; NLR, Neutrophil-to-Lymphocyte Ratio; SOFA, Sequential Organ Failure Assessment. Supplementary Table S1. Characteristics of the study groups. Categorical data are presented as *n* (%), and quantitative data as median (IQR); * Statistical comparison between groups with and without TREC data. ** Statistical comparison between groups with sequential and singular TREC measurements; *p*-values in bold are significant after FDR correction. Supplementary Table S2. The full set of qualifying variants (QVs) in the cohort of 84 patients. The QVs in each of the TREC slope quartiles in the sub-cohort of 27 patients are presented. Supplementary Table S3. Gene set enrichment analysis of genes with qualifying variants. The table includes enriched terms with a gene number per term of ≥5; Terms highlighted in red are presented in Figure 4. Supplementary Table S4. Partial correlations with initial TREC level, controlling for age. Supplementary Table S5. Enrichment analysis of immune genes related to TREC slope quartile Q1 within the Biological Process module of the Gene Ontology, as performed using Enrichr. We applied a cutoff of greater than one gene per GO term and an FDR-adjusted *p*-value of less than 0.05. Supplementary Table S6. Biological annotation of qualifying variants in immune genes for patients with extreme TREC dynamics; According to HP:0002715: Abnormality of the immune system, one from seven patients in the Q1 group and two from six patients in the Q4 group did not have qualifying variants (QVs) in immune genes.
