# Supplementary material for: Genetic Determinants of T-Cell Homeostasis in Critical Illness: An Exploratory Analysis of Immune Gene Variants and TREC Dynamics
Source: J Pers Med. 2026 May 23;16(6):278. doi: 10.3390/jpm16060278 (PMC13300954; doi:10.3390/jpm16060278)
Supplement: Supplementary file 1 [file jpm-16-00278-s001.zip › Suppl. Figures.pdf]

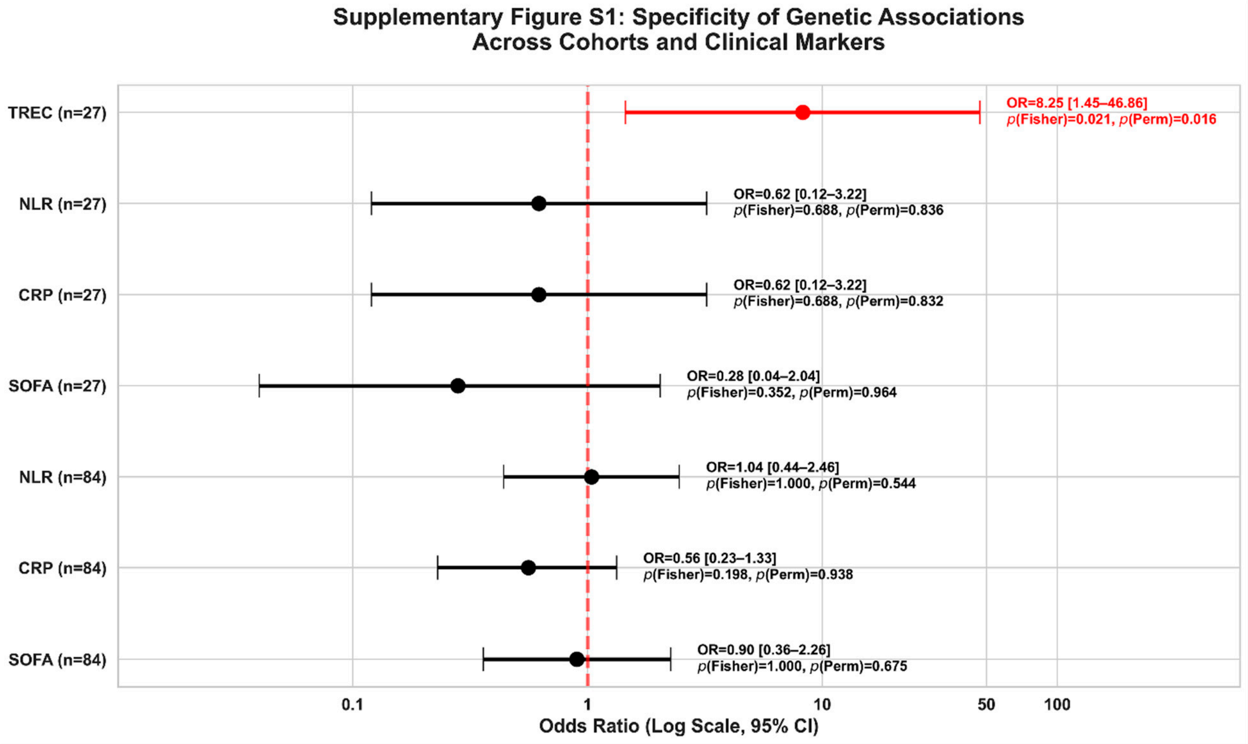

Supplementary Figure S1. Genetic analysis of qualifying variants in patients with extreme NLR, CRP (mg/L), and SOFA dynamics (slope quartile 1 vs. quartile 4) in the TREC sub-cohort of 27 patients and in the full cohort of 84 patients. Abbreviations: CRP, C-Reactive Protein; NLR, Neutrophil-to-Lymphocyte Ratio; SOFA, Sequential Organ Failure Assessment.

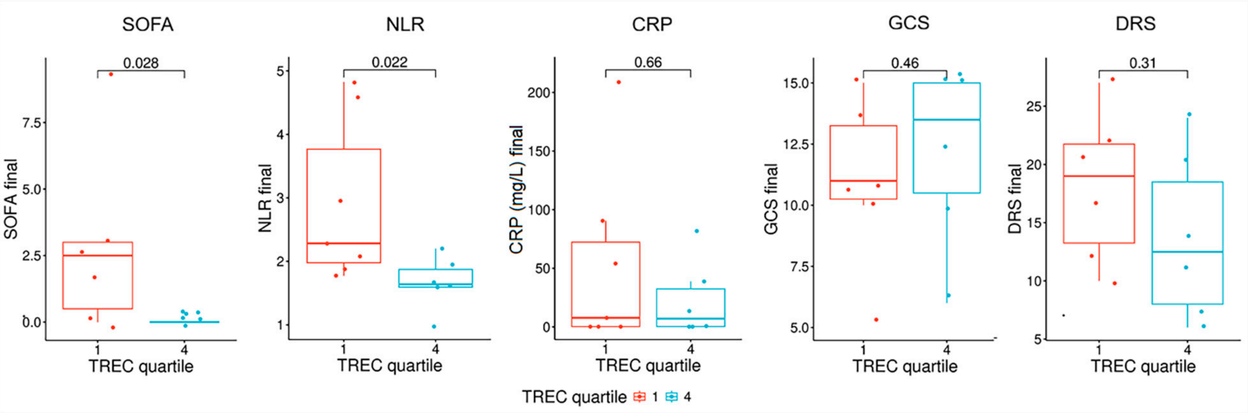

Supplementary Figure S2. Comparison of final discharge/death parameters in the five-panel analysis of Q1 vs. Q4 patients. Abbreviations: CRP, C-Reactive Protein; DRS, Disability Rating Scale; GCS, Glasgow Coma Scale; NLR, Neutrophil-to-Lymphocyte Ratio; SOFA, Sequential Organ Failure Assessment.
